# Risk of selection bias in randomized trials: further insight

**DOI:** 10.1186/s13063-016-1597-5

**Published:** 2016-10-07

**Authors:** Vance W. Berger

**Affiliations:** National Cancer Institute and University of Maryland Baltimore County, Biometry Research Group, National Cancer Institute, 9609 Medical Center Drive, Rockville, MD 20850 USA

**Keywords:** Allocation concealment, Berger-Exner test, Conditional unrestricted randomization, Maximal procedure

## Abstract

The quality of randomization is an under-appreciated facet of trial design. The present piece represents an advance in our collective understanding of how allocation concealment and randomization relate to risk of selection bias in randomized trials, and other measures are also considered. Though the overwhelming majority of the advice given is timely and correct, it is more instructive to focus on the relatively narrow sliver of advice that is incorrect (namely, that trials should not stratify by site, and that unrestricted randomization is a solution to the problem of selection bias), so it is in this context that the comments here must be understood. In no way is this intended to be a rebuttal of the excellent work we have before us. Rather, it is a refinement.

## Background

Kahan, Rehal, and Cro [1] are to be congratulated for drawing attention to a rather important problem in trial design, namely how to reduce or eliminate the risk of selection bias. The remedial methods discussed include (1) masking recruiters, (2) the use of unrestricted randomization, (3) not stratifying by site in multicenter trials, (4) avoiding permuted blocks when stratifying by site, and (5) making better use of prognostic covariates. Among the major findings are that 3 % of the trials used unrestricted randomization, 35 % did not specify how they randomized at all, and 58 % of those that stratified by site used permuted blocks. We wish to focus on these findings, and also on another opportunity to curb selection bias, namely post-trial auditing as a disincentive.

## The true nature of allocation concealment

It is stated that even with appropriate allocation concealment, prediction may still be possible. This misguided statement represents a misunderstanding of allocation concealment, and needs to be refuted since the notion is so prevalent. As one might guess from the name itself, allocation concealment means that the allocations are concealed. If they can be predicted, then we do not have appropriate allocation concealment, but, rather, have (at best) only partial allocation concealment. Cleary, allocation concealment is not a binary phenomenon [2], and its success depends on addressing *both* threats, direct observation *and* prediction. In other words, improper randomization constitutes a violation of allocation concealment [3]. These are not two distinct dimensions of trial quality, and should not be treated as such.

## Unrestricted randomization: is 3 % too much, or too little?

The authors put forth unrestricted randomization as a method to eliminate selection bias, and indeed it is. But when we bear in mind that we can prevent all carpal tunnel syndrome instantly by amputating all hands as a preventative measure, we recognize that eliminating one problem is not enough. The solution must also not introduce additional problems. Unfortunately, unrestricted randomization does precisely that, and this is why nobody has ever used it, or ever would use it in an actual trial. The 3 % is not, as suggested, too small a figure; it is, on the contrary, too large a figure.

This may sound like an audacious claim. How can we state that unrestricted randomization has never been used when the authors report that 3 % figure? In fact what *is* used when authors claim unrestricted randomization is not actually unrestricted randomization, as we shall explain. They instead use a vaguely defined variation we shall refer to as *conditional* unrestricted randomization. One major problem with unrestricted randomization is chronological bias, or the possibility of many more early patients ending up in one treatment group and many more later patients ending up in the other treatment group [4, 5]. In fact, this chronological bias is the primary reason that restricted randomization is used (as it should be) [6]. However, we shall focus instead on another issue with unrestricted randomization. Just as a normal distribution for heights means that, sooner or later, we will encounter an individual with a negative height [7], so too is it the case that with true unrestricted randomization we will, sooner or later, see an allocation sequence comprised entirely of only one treatment group.

We do not believe that any clinical trial researcher would ever, under any circumstances, accept such an unfortunate outcome. If confronted by this, they would “throw it back” and try again. But this discretion to do that calls into question just which allocation sequences would be considered admissible? We do not get around this consideration by appeal to the fact that we did not have to throw the first one back, nor by the old adage about not being able to define it but knowing it when we see it. In a world of precise definitions, this simply will not cut it. Knowledge of the sampling properties of the randomization procedure is possible only when we actually know what the randomization procedure is. So would we have accepted a split of 95:5? What about 90:10? Where exactly is the line drawn? And even if we are not only OK but also thrilled with the ideal ratio of 50:50, are we still OK with a 50:50 sequence that is segregated, with the first half of the allocations all going to the same group, and then the rest going to the other group to balance out the numbers at the end? No, we are not.

Hopefully, it is clear that “unrestricted randomization” is a procedure that nobody would ever use in an actual clinical trial and, moreover, it is sufficiently poorly defined that when it is claimed we cannot decipher what procedure actually was used. If pressed, then a researcher who claimed to use unrestricted randomization would have to come to grips with just how large an imbalance in group sizes he or she would tolerate. In fact, there is a class of randomization procedures that explicitly takes into account this maximally tolerated imbalance (MTI), namely the MTI procedures, including the big stick (essentially unrestricted randomization until the MTI is reached, at which point the allocation is forced to restore balance) [6], Chen’s procedure (a refinement of the big stick in which a biasing probability is specified so as to encourage a move towards balance without forcing it, at least until the MTI is reached) [8], and the maximal procedure (which selects randomly from among the allocation sequences that adhere to the MTI condition) [4, 5, 9]. As it turns out, these MTI procedures are not only expressed more precisely (and honestly) than unrestricted randomization, but they are also far more suitable for actual trials by virtue of controlling chronological bias and eliminating the possibility of unfortunate outcomes of the type we described earlier.

So the authors are correct in spirit. We do need randomization procedures with fewer restrictions. But the solution is not to swing the pendulum all the way to the other side. We do still need *some* restrictions, namely the MTI. But the call should be for no *additional* restrictions above and beyond this, and also for larger MTI values than those that are typically used in practice, plus dropping the requirement of terminal balance.

## Failure to specify how the trial was randomized

The authors are correct that failure to specify how the trial was randomized is simply unacceptable. And yet this occurred in 35 % of the trials evaluated. One can only imagine a conversation between a patient (P), a prescribing physician (PP), and a statistician (S):

P: Is this treatment you prescribed the best option given my condition?

PP: The evidence suggests that it is.

P: I understand that medical studies are conducted to the highest and most rigorous standards possible?

PP: Yes, they are. The stakes are too high for anything less than the best.

P: And this is why the highest level of evidence is reserved for randomized trials?

PP: Exactly.

P: Are all types of randomization equally rigorous, or are some better than others?

PP: This is a question for the statistician.

S: Some methods of randomization are better than others. The worst ones, such as permuted block randomization, can be easily deciphered and subverted.

P: I see! Well, in that case, it’s a good thing that the trials whose results have informed your decision on how to treat me did not use permuted block randomization!

S: Well, they may have. We actually don’t know if they did or didn’t.

P: I understand. I would not expect you to memorize all the details of every study. But for my peace of mind, can you please check on that when you go back to your office and have the studies available?

S: No, I happen to have the articles right here with me. It is not that I cannot remember. We do not know because the articles did not specify how they randomized.

P: So you are telling me that they may have used a valid randomization procedure, and they may not have? We have no way to know? And if they didn’t, then the trial results may bear little or no resemblance to the reality governing how effective this treatment is for my condition?

S: That is correct.

P: And instead of calling these authors to account, you just assume that they randomized correctly, and then you act accordingly in your prescribing decisions? Don’t ask, don’t tell? Even if that means that patients may then get exposed to harmful treatments?

And this is where our hypothetical dialogue ends, but I would be curious to know how real physicians might respond at this point. The enormous influence on trial quality and the reliability of the precise method of randomization cannot be overstated. The potential for misleading evidence resulting from flawed randomization methods has been well-documented [5]. And yet even given how important this information is, over one third of the trial authors could not be bothered to supply it, and over one third of the journal review teams could not be bothered to insist on it as a condition for publication. The very essence of evidence-based medicine is trust but verify (and that trust part is optional), yet consumers of medical research are put in a position of having to take it on faith that the research teams conducted rigorous research despite the fact that these very same research teams clearly were not rigorous at all in their reporting? This is not even a matter of trust. Trust would be believing the claim, but here, no claim is even offered. They do not even claim to have randomized correctly. Credibility in a system that allows for this cavalier attitude towards the lives and health of actual patients might be misplaced.

## Permuted blocks

It is pointed out [1] that 58 % of the stratified trials used permuted blocks. We can all agree that this is way too high, and the ideal proportion would be somewhere close to zero. Even one trial using permuted blocks is one too many. But what about trials that are not stratified by center? Are permuted blocks OK in these? The answer is still no. Just as the MTI procedures are far superior to unrestricted randomization by virtue of their better ability to handle chronological bias, so too are they far superior to permuted block randomization, but here due to their superior ability to control selection bias [9, 10]. The best randomization procedure for eliminating selection bias is unrestricted randomization, yet for the reasons articulated above, we are still opposed to its use in practice. Might the same argument be offered to justify using permuted block randomization even though the MTI methods are superior? No, it cannot.

The comparison of any two randomization methods, whether we are comparing unrestricted randomization to the maximal procedure or whether we are comparing blocked randomization to the big stick, must necessarily account for *both* selection bias *and* chronological bias. The fact that unrestricted randomization wins on only one of these comparisons is, as we have seen, insufficient to recommend its use, given its drawbacks on the other dimension. But that is not the case when stating that the MTI procedures are superior to blocked randomization. Here, the superiority is in an overall sense, and not just in one isolated dimension. There is no compensation by appeal to other dimensions. The MTI procedures match blocked randomization for control of chronological bias, and beat it soundly for control of selection bias, in fact in more ways than one [9]. As such, the use of permuted block randomization is indefensible.

One reviewer pointed out that permuted block randomization should be fine in masked trials, since even an occasional unmasked allocation would likely occur after the block is complete, and also for multicenter trials for which no one investigator can keep track of all allocations, since some will occur at other centers. This is, in fact, a widely held view, and likely contributes to the reluctance to switch from permuted block randomization to MTI randomization, so it does merit a thoughtful response.

First, in multicenter trials, randomization is generally stratified by center, so that in fact an investigator can keep track of how the allocation is progressing, at least at his or her center, but then this is all that is relevant for prediction anyway. Second, even if randomization were not so stratified, investigators can still predict successfully, even without certainty, based on how many patients have so far been randomized to each group. True, this can be done with MTI randomization too, but then it will be less successful than it will be with permuted blocks. Moreover, some unmasking in trials that are planned as masked can be immediate, as with injection site reactions [11]. And even if masking is retained perfectly throughout, so that selection bias of the type we consider is not an issue, there is still no benefit in using permuted block randomization. In that case, it is just as good as, but not better than, MTI randomization [9]. So we are comparing two procedures, one of which is clearly better in one situation and is just as good in the other situation, and we would not know ahead of time which situation we would be in.

This discussion makes clear that permuted block randomization should not be used. It does not, however, support the use of MTI randomization, since it would be a false dichotomy to believe that these are the only two options. Clearly, they are not. Proschan [12] discusses some other types of randomization that might be used, and, indeed, these too would be preferable to permuted block randomization. However, in other work we have found MTI randomization to be optimal, so it is these procedures that we focus on, and recommend for use in practice. Future work will extend the MTI procedures to unequal allocation, and more than two treatment groups, but for now, at least we can point out that it does seem prudent to replace permuted block randomization with MTI randomization, at least for trials with two arms and a 1:1 allocation.

## Post-trial auditing

One ideal opportunity to control selection bias was not touched upon, but should have been. It seems pretty close to research malpractice to not specify how the randomization was conducted, and it is just as bad to not formally test for selection bias after the fact. This widespread failure to even consider selection bias allows offending investigators to fly under the radar and to carry on without any fear of any real consequence. There simply is no disincentive to engage in this type of behavior. Clearly, this needs to change if trial results are to remain credible even after the public comes to understand just how easy the results are to manipulate. They will need to be assured that while it is theoretically possible to manipulate trial results, actual trials are immune because only best practices are used. Presently, we are not even close to being able to claim this.

Post-trial auditing needs to be standard and routine. It should not be triggered only when there is some basis for suspicion [13], unless the inherent vested interests the researchers have when conducting the trials themselves constitute a firm basis for suspicion, as clearly they should. The most reliable method for testing for selection bias in a randomized trial is the Berger-Exner test [14], which is based on a comparative analysis of those patients who could have been anticipated to end up in the active treatment group versus those who ended up there by chance. One can only wonder how many of the trials considered conducted this analysis, although the silence on this issue speaks volumes and we pretty much already know the answer.

## Conclusions

There is a major disconnect between the perception of medical research as a pristine beacon of hope working to save us all, and the reality of medical research as a business conducted at least partially to enrich those engage in it. Certain outcomes are more profitable, and the trials are conducted, for the most part, by the very parties who stand to gain or lose based on the trial outcomes. Moreover, these same parties with the vested interests also enjoy almost unfettered discretion to conduct the trials as they see fit, subject to some constraints but, as we have seen, constraints that still allow for discretion in deciding, among other things, to randomize properly or not. This key component of trial quality is left as a personal decision.

Improper randomization, such as permuted block randomization, invites the type of selection bias that can masquerade as a treatment effect even when the treatment in fact is no more effective than the control (or placebo). So while inertia remains a huge problem in getting researchers to upgrade their methodologies [15], it is not the *only* problem. There is more at play here, including a perverse system of incentives that works to reward researchers for using flawed research methodology. What possible incentive do researchers have to get it right when doing so will hurt their bottom line and, moreover, given that it is optional anyway? Why report honest trial results when doing so will put you at a strategic disadvantage relative to your competitors, some of whom may be using blocked randomization and any other trick they can get away with?

Clearly, self-policing does not work. It is time for an external agency to step in and clean up the mess. Only when true accountability is demanded of all medical researchers can we expect, first, better reporting of randomization methods, and second, better randomization methods. The first step has to be zero tolerance for failure to report the randomization method used in a trial, and zero tolerance for failure to audit the trial for selection bias after the fact. Short of these steps, the public has every reason to withdraw whatever trust it has left in the medical establishment.

## Abbreviations

MTI: Maximally tolerated imbalance
P: Patient
PP: Prescribing physician
S: Statistician
